# Weight, height, body mass index and risk of breast cancer in postmenopausal women: a case-control study

**DOI:** 10.1186/1471-2407-8-278

**Published:** 2008-09-30

**Authors:** Ali Montazeri, Jila Sadighi, Faranak Farzadi, Farzaneh Maftoon, Mariam Vahdaninia, Mariam Ansari, Akram Sajadian, Mandana Ebrahimi, Shahpar Haghighat, Iraj Harirchi

**Affiliations:** 1 Iranian Institute for Health Sciences Research (IHSR), Tehran, Iran; 2 Iranian Centre for Breast Cancer (ICBC), Tehran, Iran; 3 Public Health and Health Policy, Division of Community-Based Sciences, University of Glasgow, Glasgow, Scotland, UK

## Abstract

**Background:**

Many women in Iran have a relatively high body mass index. To investigate whether the condition contributes to excess breast cancer cases, a case-control study was conducted to assess the relationships between anthropometric variables and breast cancer risk in Tehran, Iran.

**Methods:**

All incident cases of breast cancer in the Iranian Centre for Breast Cancer (ICBC) were identified through the case records. Eligible cases were all postmenopausal women with histological confirmed diagnosis of breast cancer during 1996 to year 2000. Controls were randomly selected postmenopausal women attending the ICBC for clinical breast examination during the same period. The body mass index (BMI) was calculated based on weights and heights as measured by the ICBC nursing staff. Both tests for trend and logistic regression analysis were performed to calculate odds ratios and 95% confidence intervals as measures of relative risk.

**Results:**

In all, 116 breast cancer cases and 116 controls were studied. There were no significant differences between cases and control with regard to most independent variables studied. However, a significant difference was observed between cases and controls indicating that the mean BMI was higher in cases as compared to controls (P = 0.004). Performing logistic regression analysis while controlling for age, age at menopause, family history of breast cancer and parity, the results showed that women with a BMI in the obese range had a three fold increased risk of breast cancer [odds ratio (OR) = 3.21, 95% confidence interval (CI): 1.15–8.47].

**Conclusion:**

The results suggest that obesity in postmenopausal women could increase risk of breast cancer and it merits further investigation in populations such as Iran where it seems that many women are short in height, and have a relatively high body mass index.

## Background

Although anthropometric characteristics have been evaluated as possible determinants of breast cancer risk [1-3], studies on the association of obesity with breast cancer risk in Western women have provided contradictory results. While postmenopausal women show a positive association between increased body mass and breast cancer risk, the association is found to be negative in pre-menopausal breast cancer [4,5]. In the New York University Women's Health Study the authors concluded that excessive body weight increases breast cancer risk in postmenopausal women. On the contrary, in pre-menopausal women, excessive body weight may be protective among women who have a lower-body type of fat accumulation [6].

Studies suggest that timing of weight gain in an individual may be more relevant to breast cancer risk than weight gain at any age [7]. In other words it is argued that being overweight before the age of 18 years is associated with a reduced risk of breast cancer in pre-menopausal women, while being overweight after the age of 18 years is associated with a higher risk of postmenopausal but not of pre-menopausal breast cancer [8,9]. Similar observations were reported in several case-control studies [10,11]. A population-based case-control study in Sweden indicated that women who had gained 30 Kg or more since age 18 had a twofold risk of breast cancer compared with those who had maintained their weight unchanged [11]. Overall, the evidence suggests that postmenopausal women who are overweight or obese have an increased relative risk of breast cancer, for instance ranging from 1.23 (CI: 1.00–1.59) [12] to 2.52 (95% CI: 1.62–3.93) [13].

However, not enough is known about the relationship between anthropometric variables and risk of breast cancer in Asian populations. An international study reported that in Asian populations with low breast cancer risk, both pre- and post-menopausal obesity was associated with a higher risk of breast cancer [14]. Similarly a recent study from India indicated that increased body mass index, waist size and hip size were risk factors for breast cancer both in pre- and postmenopausal women [15]. However, a study of pre-menopausal Vietnamese and Chinese women found that body mass index was not significantly related to breast cancer risk [16].

Only one study has reported on associations between body mass index and breast cancer risk in Iran [17]. Studies concerning obesity in Iran indicate that excess body weight is common among women, and more Iranian women than men present with overweight and abdominal obesity [18]. In addition, evidence from Iran suggests that lack of physical activity and sedentary lifestyle are responsible for the emerging obesity [19], although the role of nutrition should not be neglected.

This paper aims to examine the relationship between anthropometric variables and risk of breast cancer in postmenopausal women in Tehran, Iran where it seems that many women have a relatively high body mass index.

## Methods

A retrospective case-control study was conducted to assess the relationships between anthropometric variables and breast cancer risk in Tehran, Iran. All incident cases of breast cancer in the Iranian Centre for Breast Cancer (ICBC) were identified through the case records. Eligible cases were all postmenopausal women with histologically confirmed diagnosis of breast cancer (both in situ and invasive disease) during 1996 to year 2000 and women with a previous breast cancer diagnosis were excluded. Controls were randomly selected from postmenopausal women attending the ICBC for clinical breast examination during the same period. Menopausal status was defined when there have been no menses for a year (natural menopause). Women with bilateral oophorectomy, hysterectomy, or who reported use of postmenopausal hormones were excluded from the study. Data on demographic, medical history and reproductive characteristics of breast cancer cases and controls were extracted from case records. The variables of interest were age, marital status, educational status, family history of breast cancer, age at menarche, parity, age at first live birth, number of live births, age at menopause, previous use of oral contraceptives, weight and height at diagnosis. The body mass index was calculated based on heights and weights as measured by the ICBC nursing staff [BMI = weight (kg)/height (m)^2^]. To avoid measurement error one of the authors carried out duplicate measurements at the same time and the two measurements were identical as noted by one of us. Based on the BMI, women were grouped into different categories as recommended by the WHO: normal range (BMI = 18.5–24.9 kg/m^2^), overweight (BMI = 25–29.9 kg/m^2^), and obese (BMI ≥ 30 kg/m^2^) [20].

The sample size was based on an assumption that at least 25% of breast cancer cases and 10% of women in the control group would be obese. As such, a study with a sample of 224 women (112 in each group) would have a power of 80% to estimate an odds ratio of 3 at 95 percent confidence level [21]. The actual sample studied here was 232 postmenopausal women (116 per each group).

Tests for trend were performed to calculate odds ratio and 95% confidence intervals as measures of relative risk. The test calculates Chi-square for trend, the associated P value and the odds ratio for each category compared with the reference category [21]. T-test was carried out to compare the BMI, height, and weight mean differences between cases and controls. In addition, logistic regression analysis was performed to estimate odds ratios for developing breast cancer while adjusting for age, age at menopause, family history of breast cancer and parity.

The ICBC board approved the study and all patients were asked for consent to use their information for the study. In fact, since there was no interventions involved a member of the ICBC called each patient (116 cases and 116 controls) to ask for permission to use their information to be reviewed. All patients gave oral consent.

## Results

In all, 116 breast cancer cases and 116 controls were studied. The characteristics of cases and controls are shown in Table 1. The mean age for cases was 54.6 (SD = 6.8) while it was 52.7 (SD = 6.8) for controls (P = 0.03). With regard to the BMI categories there was a significant difference between cases and controls (Table 2). In addition, when the BMI mean difference between cases and controls was assessed, a significant difference was observed indicating that the mean BMI was higher in cases as compared to controls (P = 0.004). Further analysis of data indicated that breast cancer cases were significantly shorter than controls (P = 0.00003). With regard to the recent weight, although the breast cancer cases tended to be heavier, there was no significant difference between cases and controls (Table 2).

**Table 1:** Demographic, medical history, and reproductive characteristics of breast cancer cases and controls

	**Cases (n = 116)** **No. (%)**	**Controls (n = 116)** **No. (%)**	**Odds ratio (95% CI)**	**χ^2^for trend (P)**
**Age (years)**				
< 50	28 (24)	43 (37)	0.6 (0.3–1.4)	
50–54	43 (37)	33 (28)	1.3 (0.6–2.0)	
55–59	25 (22)	24 (21)	1 (ref.)	
60 ≥	20 (17)	16 (14)	1.2 (0.5–2.9)	
				1.0 (0.3)
Mean (SD)	54.6 (6.8)	52.7 (6.8)		0.03*
**Marital status**				
never married	7 (6)	4 (4)	1.0 (ref.)	
married	86 (74)	84 (72)	0.7 (0.2–2.5)	
widowed/divorced	23 (20)	28 (24)	0.6 (0.1–2.3)	
				0.7 (0.4)
**Educational status**				
illiterate	22 (19)	29 (25)	1.0 (ref.)	
primary education	36 (31)	31 (27)	1.5 (0.7–3.3)	
secondary education	38 (33)	42 (36)	1.2 (0.6–2.6)	
higher education	20 (17)	14 (12)	1.9 (0.7–4.8)	
				0.9 (0.4)
**Family history of breast cancer**				
none	88 (76)	94 (81)	1.0 (ref.)	
first or second degree	28 (24)	22 (19)	1.4 (0.2–9.7)	
				0.7 (0.4)
**Age at menarche (years)**				
< 13	28 (24)	18 (16)	1.0 (ref.)	
13	36 (32)	33 (28)	0.8 (0.3–1.7)	
14	26 (22)	37 (32)	0.5 (0.2–1.1)	
14 >	26 (22)	28 (24)	0.6 (0.3–1.4)	
				2.2 (0.1)
**Parity**				
nulliparous	11 (9)	7 (6)	1.0 (ref.)	
multiparous	105 (91)	109 (94)	0.7 (0.2–1.9)	
				0.7 (0.4)
**Age at first live birth (years)**				
14–19	36 (34)	46 (42)	1.0 (ref.)	
20–24	44 (42)	43 (40)	1.3 (0.7–2.4)	
25–29	17 (16)	13 (12)	1.7 (0.7–4.0)	
30 ≥	8 (8)	7 (6)	1.7 (0.5–5.2)	
				1.6 (0.2)
**Number of live births**				
none	11 (9)	7 (6)	1.0 (ref.)	
1–2	27 (24)	33 (28)	0.6 (0.2–1.8)	
3–4	45 (39)	32 (28)	0.9 (0.3–2.9)	
> 4	33 (28)	44 (38)	0.5 (0.2–1.5)	
				0.8 (0.4)
**Age at menopause (years)**				
< 45	29 (25)	40 (35)	1.0 (ref.)	
45–50	59 (51)	53 (45)	1.6 (0.8–3.0)	
> 50	28 (24)	23 (20)	1.7 (0.8–3.7)	
				1.9 (0.2)
**Previous use of oral contraceptives**				
none	73 (63)	75 (65)	1.0 (ref.)	
yes	43 (37)	41 (35)	1.1 (0.6–2.0)	
				0.1 (0.8)

* Derived from t-test.

**Table 2:** Body mass index, height and weight in cases and controls

	**Cases (n = 116)** **No. (%)**	**Controls (n = 116)** **No. (%)**
**Body mass index (kg/m**^2^**)**		
18.5–24.9	23 (20)	40 (35)
25–29.9	51 (44)	47 (40)
≥ 30	42 (36)	29 (25)
Test for trend	χ^2 ^= 7.1, P = 0.03	
Mean BMI (SD)	27.9 (3.9)	26.3 (4.3)
T-test	t = 2.9, P = 0.004	
Height* (Cm)		
≤ 157	61 (53)	31 (27)
158–160	29 (25)	34 (29)
161–164	19 (16)	23 (20)
≥ 165	7 (6)	28 (24)
Test for trend	χ^2 ^= 23.1, P = 0.00003	
Mean height (SD)	158.5 (4.1)	161.3 (5.2)
T-test	t = 4.5, P < 0.0001	
Weight* (Kg)		
≤ 62	24 (21)	30 (26)
63–67	25 (22)	28 (24)
68–75	35 (30)	30 (26)
≥ 76	32 (27)	28 (24)
Test for trend	χ^2 ^= 1.49, P = 0.68	
Mean weight (SD)	69.5 (7.6)	68.0 (7.4)
T-test	t = 1.4, P = 0.14	

* The grouping for height and weight are based on approximate quartile values of the control population.

The results obtained from multiple logistic regression analysis are shown in Table 3 indicating that overweight and obese women were at higher risk of developing breast cancer (OR = 2.53, and 3.21 respectively). The analysis showed that height significantly contributed to the observed differences.

**Table 3:** The odds ratios for postmenopausal breast cancer obtained from unconditional logistic regression analysis

	**OR (95%CI)**	**P**
**Model 1**		
***a. BMI (kg/m^2^)***		
18.5–24.9	1.0 (ref.)	
25–29.9	2.53 (1.20–5.35)	0.01
≥ 30	3.21 (1.15–8.47)	0.02
*Age*	1.03 (0.95–1.10)	0.44
*Age at menopause*	0.98 (0.93–1.03)	0.43
*Family history of breast cancer*		
first or second degree	1.36 (0.71–2.63)	0.35
*Parity*		
multiparous	0.48 (0.17–1.37)	0.17
***b. Height (cm)***		
≤ 157	1.0 (ref.)	
158–160	0.15 (0.10–0.40)	< 0.0001
161–164	0.10 (0.19–0.32)	< 0.0001
≥ 165	0.08 (0.05–0.21)	< 0.0001
*Age*	0.94 (0.87–1.02)	0.13
*Age at menopause*	0.95 (0.91–1.00)	0.09
*Family history of breast cancer*		
first or second degree	1.36 (0.68–2.73)	0.38
*Parity*		
multiparous	0.20 (0.06–0.65)	0.01
***c. Weight (Kg)***		
≤ 62	1.0 (ref.)	
63–67	1.02 (0.20–2.09)	0.67
68–75	1.13 (0.40–2.43)	0.54
≥ 76	1.26 (0.42–2.82)	0.47
*Age*	1.08 (1.00–1.17)	0.03
*Age at menopause*	0.98 (0.93–1.03)	0.44
*Family history of breast cancer*		
first or second degree	1.35 (0.71–2.59)	0.36
*Parity*		
multiparous	0.45 (0.16–1.32)	0.15
**Model 2**		
***a. BMI (kg/m^2^)***	1.15 (1.03–1.30)	0.02
*Age*	0.99 (0.92–1.08)	0.99
*Age at menopause*	0.97 (0.93–1.02)	0.32
*Family history of breast cancer*		
first or second degree	1.46 (0.76–2.81)	0.25
*Parity*		
multiparous	0.40 (0.14–1.14)	0.08
***b. Height (cm)***	0.73 (0.65–0.82)	< 0.0001
*Age*	0.95 (0.88–1.02)	0.16
*Age at menopause*	0.97 (0.92–1.02)	0.30
*Family history of breast cancer*		
first or second degree	1.43 (0.71–2.86)	0.31
*Parity*		
multiparous	0.34 (0.11–1.04)	0.06
***c. Weight (Kg)***	1.00 (0.93–1.06)	0.88
*Age*	1.07 (0.99–1.16)	0.08
*Age at menopause*	0.98 (0.93–1.03)	0.47
*Family history of breast cancer*		
first or second degree	1.38 (0.73–2.63)	0.32
*Parity*		
multiparous	0.47 (0.17–1.31)	0.14

**Model 1: **BMI, height, and weight independently were entered into the model as categorical data while adjusted for age, age at menopause, family history of breast cancer and parity.

**Model 2: **BMI, height, and weight independently were entered into the model as continuous data while adjusted for age, age at menopause, family history of breast cancer and parity.

## Discussion

Overweight and obesity are common health conditions and their prevalence is increasing globally. The World Health Organization recently called for the continuous monitoring of BMI to assess the trends in obesity in populations across time [22]. It is well known that excess weight is associated with increased incidence of particular diseases and certain cancers and those associations of excess weight with overweight and obesity-related morbidity may differ among racial and ethnic groups [23]. Although the result of this study with a small sample size could not be generalized, the findings indicated that obesity was a risk factor for developing breast cancer in postmenopausal women in Iran. The obese women showed about threefold risk of breast cancer (OR = 3.21) compared to women with a normal body mass index. It is argued that it is unclear whether this relationship reflects a causal role of obesity during childhood and adolescence, of weight gain during adult life, or of adult obesity per se [11]. Recent studies suggest that relation between breast cancer risk in young women and body weight at different ages is complex and that the risk reduction with adult weight gain is confined to less aggressive cancers [24]. In addition, as indicated by some researchers the critical pathways for understanding the relation between obesity and breast cancer risk are likely to be depend on two points in time: at menarche and menopause; time when some metabolic factors occur and women would experience some physiological (hormonal) changes in their body [25,26].

Weight gain and obesity not only are risk factors for breast cancer, but also contribute to the diagnosis process. A study from Geneva Cancer Registry, Switzerland compared diagnostic characteristics of obese with non-obese breast cancer patients. The findings showed that obese patients presented significantly more often with advanced stage disease, experienced more surgical delay and prolonged hospital stay after surgery. The authors speculated that the more advanced stage at diagnosis could be due to the larger breast size of obese women, with more fatty tissue, which can lead to difficulties in palpating the primary tumor and the axillary lymph nodes. Also, obese women may be more reluctant to undergo physical examination because of embarrassment about their weight. They suggest there is a need to educate women and doctors that self-examination and clinical examination may be less reliable in obese women, and to think of strategies to prevent advanced disease at diagnosis for this growing group of patients [27].

There are two measures which are significant to body mass index and its relation to breast cancer risk: weight and height. The strength of this study relates to the fact that all heights and weights were measured by the ICBC nursing staff rather than relying on self-reported weights and heights by the cases and controls. Anthropometric studies usually rely on self-reported measures and the evidence suggests that obese women tend to underestimate their weight gain as compared to lean adolescents [28]. In contrast, this study was limited due to the fact that weight was measured at diagnosis. Weight at diagnosis may not reflect usual weight before disease onset since cases may have recently lost weight as a result of their breast cancer, or gained weight due to immobility from feeling badly.

It is argued that adult weight gain is a more important risk factor for breast cancer than BMI because body mass gained after young age is mainly adipose tissue (that increases the risk of breast cancer), while BMI is determined by fat tissue and lean body mass in combination (that might not be responsible for increased risk of breast cancer). Therefore, weight gain might be a better measure of adult obesity than BMI [8,29] or body fat mass and fat free mass components of BMI are found to be more discriminant factors for breast cancer incidence risk than the commonly used BMI [30,31]. However, it has not been shown that reducing body weight can lower breast cancer risk and this need to be studied further [32].

In this study the breast cancer cases were shorter in height than controls and it seems that the height was the most significant factor that contributed to increased breast cancer risk in obese women (Table 3). The findings of this study are challenging and were different from most studies that support a modest association between height and breast cancer risk [33]. In fact, the association observed between breast cancer risk and height in this study is in the opposite direction of what has been previously observed in other populations. The Netherlands cohort study on diet and cancer found that women with a higher height had an increased risk of breast cancer compared with shorter women in height [34]. Also height was found to be a strong predictor of breast cancer risk with comparable effects in both pre-menopausal and postmenopausal women [35]. It is argued that several factors including genetics, nutrition, diet, and hormone levels all influence height. A study from Italy has reported that no relationship was observed between the risk of breast cancer among tallest women compared with the shortest [36]. Similarly studying white women and comparing 5,358 newly diagnosed breast cancer cases and 4,555 controls there was no association between height and risk of breast cancer [37].

Finally, this study, as many investigations, showed elevated odds ratios for breast cancer associated with a number of known risk factors such as family history of breast cancer, younger ages at menarche, and older ages at first birth (Table 1). However, since the sample sizes were small, statistical significance was not achieved. Further investigations on the topic with larger sample sizes are recommended.

## Conclusion

This study provided data on the fact that obesity could increase breast cancer risk in postmenopausal women in Iran. It seems that there is need to publicize obesity as an important risk factor for breast cancer in Iran. Indeed, this requires encouraging Iranian women to do more exercise and follow healthy diets.

## Abbreviations

ICBC: Iranian Center for Breast Cancer; Kg: Kilogram; m^2^: Square meter; Cm: Centimeter; BMI: Body Mass Index; OR: Odds Ratio; CI: Confidence Interval.

## Competing interests

The authors declare that they have no competing interests.

## Authors' contributions

AM was the main investigator, analyzed the data and wrote the paper. JS, FF, and FM contributed to the study design and analysis. MV contributed to the study design and helped in writing the final draft. MA, and AK collected the data. ME, SH and IH contributed to the data collection and patient recruitments for the study. All authors read and approved the final version of the manuscript.

## Pre-publication history

The pre-publication history for this paper can be accessed here:


